# Lead halide perovskite for efficient optoacoustic conversion and application toward high-resolution ultrasound imaging

**DOI:** 10.1038/s41467-021-23788-4

**Published:** 2021-06-07

**Authors:** Xinyuan Du, Jiapu Li, Guangda Niu, Jun-Hui Yuan, Kan-Hao Xue, Mengling Xia, Weicheng Pan, Xiaofei Yang, Benpeng Zhu, Jiang Tang

**Affiliations:** 1 Wuhan National Laboratory for Optoelectronics, School of Optical and electronic information, Huazhong University of Science and Technology, Wuhan, China; 2 State Key Laboratory of Transducer Technology, Chinese Academy of Sciences, Shanghai, China

**Keywords:** Ultrasound, Sensors and biosensors, Imaging techniques

## Abstract

Lead halide perovskites have exhibited excellent performance in solar cells, LEDs and detectors. Thermal properties of perovskites, such as heat capacity and thermal conductivity, have rarely been studied and corresponding devices have barely been explored. Considering the high absorption coefficient (10^4^~10^5^ cm^−1^), low specific heat capacity (296–326 J kg^−1^ K^−1^) and small thermal diffusion coefficient (0.145 mm^2^ s^−1^), herein we showcase the successful use of perovskite in optoacoustic transducers. The theoretically calculated phonon spectrum shows that the overlap of optical phonons and acoustic phonons leads to the up-conversion of acoustic phonons, and thus results in experimentally measured low thermal diffusion coefficient. The assembled device of PDMS/MAPbI_3_/PDMS simultaneously achieves broad bandwidths (−6 dB bandwidth: 40.8 MHz; central frequency: 29.2 MHz), and high conversion efficiency (2.97 × 10^−2^), while all these parameters are the record values for optoacoustic transducers. We also fabricate miniatured devices by assembling perovskite film onto fibers, and clearly resolve the fine structure of fisheyes, which demonstrates the strong competitiveness of perovskite based optoacoustic transducers for ultrasound imaging.

## Abstract (web summary)

Lead halide perovskites are widely used e.g. in solar cells and LEDs, but devices based on thermal properties have received little attention. Here, the authors take advantage of the thermal properties to fabricate an optoacoustic transducer with both broad bandwidth and high conversion efficiency.

## Introduction

Lead halide perovskites have recently emerged as attractive optoelectronic semiconductors considering their large absorption coefficient, low defect density, long carrier lifetime, and convenient fabrication process^1–3^. These properties benefit the excellent device performance in solar cells^4,5^, light-emitting diodes^6,7^, photodetectors^8^, and radiation detectors^9,10^. Solar cells based on perovskites have achieved a certified power conversion efficiency of 25.2%, close to single-crystal silicon solar cells (https://www.nrel.gov/pv/cell-efficiency.html).

Another extraordinary character of lead halide perovskite, which has received limited attention despite potential applications, is the thermal properties, such as low thermal conductivity and small specific heat capacity. Pisoni and coworkers report the low thermal conductivity of CH_3_NH_3_PbI_3_ as 0.5 W m^−1^ K^−1^ (ref. ^11^). The recent studies find that there exists strong coupling of optical phonons and acoustic phonons in metal halide perovskites, and the strong coupling enables the up-transition of acoustic phonons, while acoustic phonons are responsible for the thermal transport. Above behavior results in short acoustic phonon lifetime (picoseconds), corresponding to nanometer mean free paths and indicating that acoustic phonons are unable to dissipate heat efficiently^12,13^. The rather small thermal conductivity of perovskites, combined with its low specific heat capacity (296–326 J kg^−1^ K^−1^), as well as high absorption coefficient (10^4^–10^5^ cm^−1^), are key features for application in optoacoustic transducer, which has never been reported.

Optoacoustic transducer can provide ultrasound pulses, and has broad applications ranging from biomedical imaging, therapeutic ablation, brain modulations to nondestructive testing^14–17^. Compared to traditional piezoelectric ultrasound transducers (large amounts of cabling and electromagnetic interference), optoacoustic transducers utilize lasers as the driving source instead of electricity, avoiding the complexity of electronic component assembly, and fiber-optic transmitters even allow the interventional cardiology applications^18–21^. Optoacoustic transducers rely on composites, with one responsible for light absorption and the other for thermal expansion, for which polydimethylsiloxane (PDMS) is exclusively used as thermal expansion layer considering its high thermal expansion (*β* = 0.92 × 10^−3^ K^−1^), optical transparency, which enables the use of visible lasers for excitation and comparable acoustic impedance to water that reduces the ultrasound loss at the interface^22^. For light absorption, carbon materials, including candle soot particles, carbon nanotubes (CNTs) and carbon nanofibers, have been widely utilized due to the large absorption coefficients and low heat capacity^18,23,24^. The state-of-the-art optoacoustic transducer utilizes the composites of CNTs and PDMS, achieving a −6 dB bandwidth of 39.8 MHz, the peak frequency of 28.5 MHz, and ultrasound peak-to-peak amplitude of ~2.72 MPa (Table 1)^18,23–26^. The above acoustic pressure and bandwidth still lags behind the traditional piezoelectric transducers. Thus, the major challenge for optoacoustic transducers is to simultaneously achieve broad bandwidths and high acoustic pressure, which are two determinative criteria for high-resolution ultrasound imaging.

**Table 1:** Performance summary of representative optoacoustic transducers.

Optical absorbing materials	Center frequency (MHz)	−6 dB bandwidth (MHz)	Conversion efficiency (×10^−2^)	Positive/negative acoustic pressure (MPa)	Reference
Cr	—	—	0.042	+1.82	^26^
Carbon nanofibers	3	7.84	0.166	+13/−1	^23^
Candle soot particles	10	21	0.441	+4.8/−1	^24^
Carbon nanotubes	28.5	39.8	—	+1.36/−1.36	^18^
Carbon nanotubes + Au	11.8	21.1	2.74	+33.6/−10	^25^
MAPbI_3_	29.2	40.8	2.97	+15/−10	This work

As we claim previously, metal halide perovskites are promising candidates to substitute the traditional carbon materials. Upon utilizing perovskite in optoacoustic transducers, its small thermal conductivity could decrease the loss rate of local heat, enhance the local temperature within the absorber layers, and thus increase the thermal conduction between absorber layer and PDMS^27^. Based on the above analysis, we demonstrate the composite of MAPbI_3_ and PDMS as optoacoustic transducers with broad bandwidth and high conversion efficiency. We experimentally measured the specific heat capacity of MAPbI_3_ as ~308 J kg^−1^ K^−1^, much lower than carbon materials. The theoretically calculated phonon spectrum indicates that the overlap of optical and acoustic phonons leads to the up-conversion of acoustic phonons, and thus results in experimentally measured low thermal diffusion coefficient of 0.145 mm^2^ s^−1^. At last, the assembled device of PDMS/MAPbI_3_/PDMS achieves a high acoustic pressure of 24.89 MPa (laser energy: 3 mJ pulse^−1^). More importantly, the −6 dB bandwidth reaches 40.8 MHz, and the optoacoustic conversion efficiency is 2.97 × 10^−2^, while above two parameters are the record values for optoacoustic transducers. We also utilized such device onto fibers and achieved high-resolution ultrasound imaging of fisheyes.

## Results

### Principle of the optoacoustic transducer

**Fig. 1:**
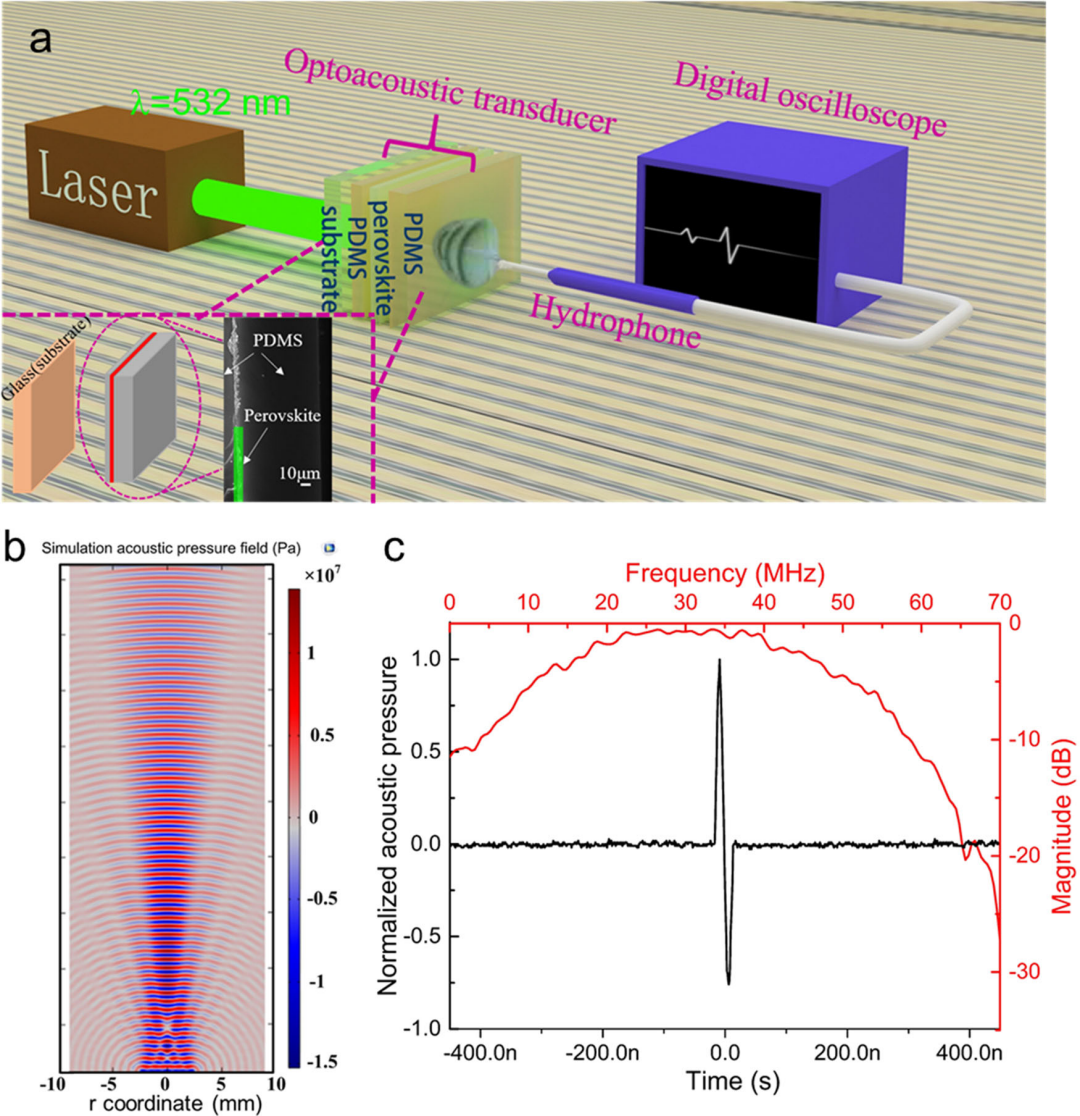
Perovskite-based optoacoustic transducer. **a** Schematic illustration of the optoacoustic transducers and the characterization system. The inset is the device structure and the cross-sectional scanning electron microscope (SEM) image of the transducer. **b** The simulated distribution of the acoustic field. **c** Experimentally measured acoustic wave (black curve) and spectrum (red curve) of the as-prepared optoacoustic transducer.

Figure 1a is the schematic illustration of the optoacoustic transducers and the characterization system. The laser-generated ultrasound of the optoacoustic transducer is detected by a hydrophone and readout by an oscilloscope. The inset of Fig. 1a shows the cross-sectional scanning electron microscope (SEM) image of the optoacoustic transducer. PDMS and MAPbI_3_ were sequentially labeled in the image. The MAPbI_3_ thin film has a thickness of 323 nm, absorbs and transfers visible photons into thermal energy. Figure 1b is the simulated distribution of acoustic field for the transducer through COMSOL Multiphysics (see Supplementary Note 1 and Supplementary Fig. 1). It can be known that the maximum acoustic pressure of COMSOL simulation results is ~17 MPa (positive acoustic pressure), the experimental results show that the maximum sound pressure is ~15 MPa. The difference may be due to the neglect of the sound attenuation in the water and different axial positions. The acoustic field is divided into near field and far field. The acoustic pressure in the far field gradually decays in the axial, and satisfies the character of approximate Gaussian distribution in the transverse. The sound beam width in the near field is closely equal to the width of the transducer (5 mm), the sound beam width in the far field is greater than the width of the transducer (5 mm).

Figure 1c shows that the central frequency and −6 dB bandwidth of the ultrasound wave is 29.2 and 40.8 MHz respectively, which is the largest among all reported optoacoustic transducer, with details summarized in Table 1. When the laser energy is 3 mJ pulse^−1^, the peak-to-peak value of acoustic pressure is 24.89 MPa and the optoacoustic conversion efficiency is 2.97 × 10^−2^ (see Supplementary Fig. 2 and Eq. (S1)). As shown in Table 1, the conversion efficiency is also the highest value for all kinds of optoacoustic transducers, better than candle soot particles/PDMS composite (0.441 × 10^−2^), CNTs/PDMS composite (0.959 × 10^−2^), and CNTs/Au/PDMS (2.74 × 10^−2^). In practical applications, we have to consider the stability of the perovskite film since the transducer is immersed in water. As shown in Supplementary Figs. 2 and 3, the transmittance (3.14% at 532 nm) of the optoacoustic transducer remains unchanged after soaking in water for 5 h. Such good stability benefits from the compact PDMS layer on the perovskite film.

### Mechanism analysis of the optoacoustic transducer

**Fig. 2:**
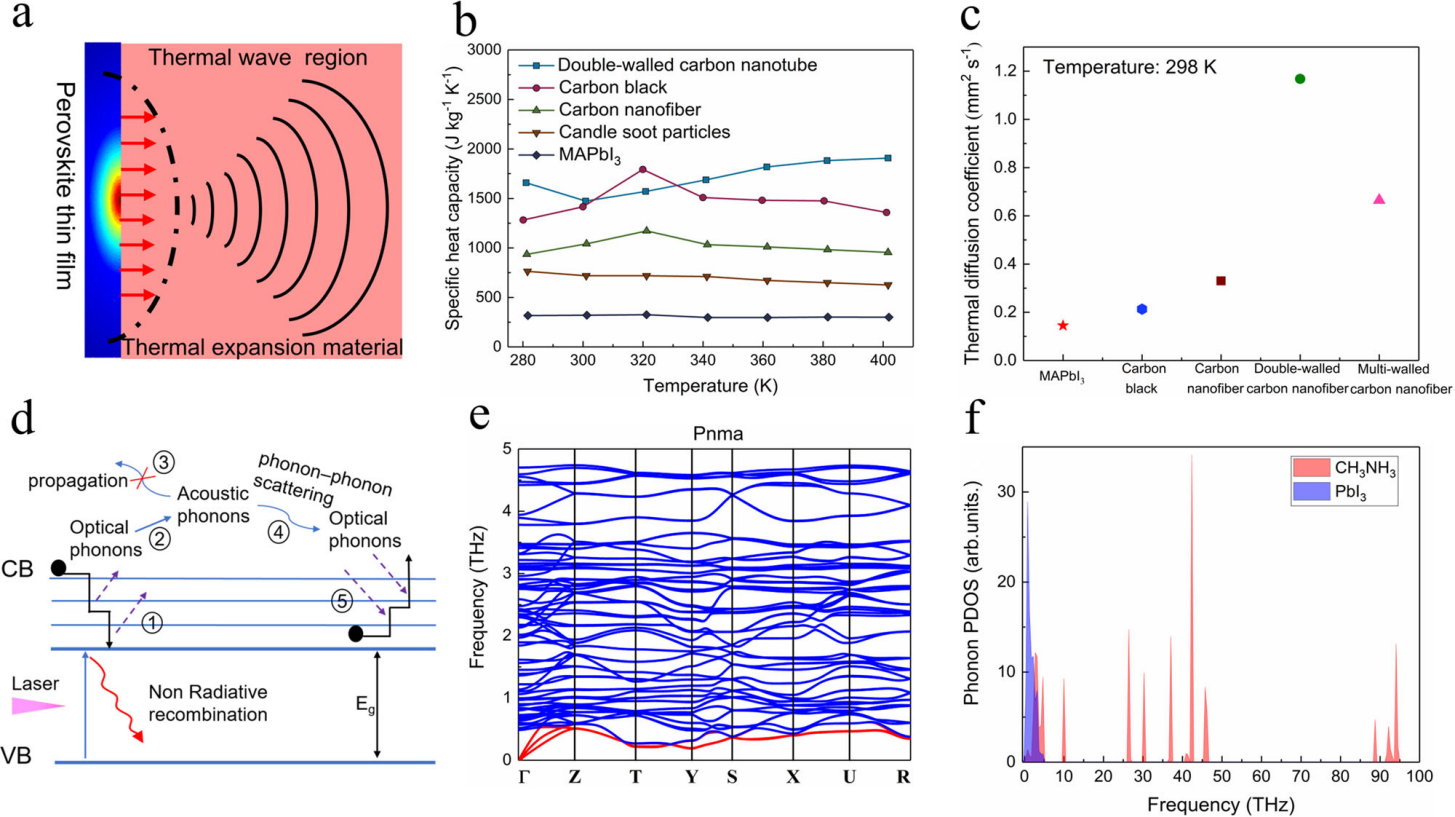
The analysis of thermal properties of MAPbI_3_. **a** The mechanism of the optoacoustic transducers. The blue rectangle is the perovskite film and the pink part represents the thermal expansion material. **b** Measured specific heat capacity of perovskites and other representative absorbers. The double-walled carbon nanotube, carbon black, carbon nanofiber, candle soot particles, and MAPbI_3_ are labeled as cyan rectangle, red circle, light green triangle, the brown inverted triangle, and blue diamond, respectively. **c** Measured thermal diffusion coefficient of different light absorption materials. The red star, blue hexagon, dark red rectangle, green circle, and pink triangle represent MAPbI_3_, carbon black, carbon nanofiber, double-walled carbon nanotube, and multi-walled carbon nanotube, respectively. **d** Schematic illustration of the heat generation process within MAPbI_3_. Here, the acronyms of CB and VB represents conduction band and valence band, respectively. The physical meaning of vertical axis is the energy levels. Specifically, the arrows of ①–⑤ represent the process of carrier relaxation. ①: The generation of optical phonons by the relaxation of high energy electrons in the conduction band; ②: The excited optical phonons decay to acoustic modes; ③: the propagation of acoustic phonons within the crystal lattice; ④: The phonon up-transition process (acoustic phonons → optical phonons) due to the energy overlap between the two modes; ⑤: The charge carriers are reheated by the recycled thermal energy. **e** Calculated phonon spectrum of MAPbI_3_. The blue curve represents optical phonon spectrum and the red curve represents acoustic phonon spectrum. **f** Density of states of the phonon spectrum.

As shown in Fig. 2a, the optoacoustic conversion generally consists of three steps: (1) light absorption by the perovskite layer, (2) the temperature rises at the interface between MAPbI_3_ and PDMS, and (3) thermal expansion of PDMS and generation of the ultrasound wave^26^.

The absorption coefficient ($\alpha$) of MAPbI_3_ is 6.7 μm^−1^ toward the incident wavelength of 532 nm (calculated by Supplementary Fig. 2), while that of the commonly used absorber layer for optoacoustic transducers, candle soot particles, is only 1 μm^−1^ (ref. ^27^). The derived thickness required to absorb 90% of the incident light is 340 nm and 2.3 μm for MAPbI_3_ film and candle soot particles, respectively. The decreased thickness of absorber layer is beneficial for the thermal confinement and efficient thermal conduction between absorber layer and PDMS layer.

Afterward, the temperature of MAPbI_3_ layer would increase, and the thermal conduction between MAPbI_3_ and PDMS results in the temperature rise of PDMS. At last, the PDMS layer is thermally expanded and contracted, following the light pulse, producing ultrasound waves. The generated acoustic pressure is expressed by the following equation^28^:

1 $${P}_{0}=\nu \beta \Delta T$$

where *P*_0_ is the peak acoustic pressure, *β* is the volume thermal expansion coefficient of PDMS (9.6 × 10^−4^ K^−1^)^29^, *ν* is the bulk modulus of PDMS (0.8–10 MPa)^30^, and Δ*T* is the temperature change. The theoretical acoustic pressure values could be estimated by Eq. (1), while the detailed process is documented in Supplementary Note 2. Under the laser excitation (3 mJ pulse^−1^), the theoretical acoustic pressure (16.16 MPa) is close to the experimental result (14.52 MPa), verifying the correctness of our model.

Based on the assumption of local thermal equilibrium, the temperature change (Δ*T*) at the interface between perovskite and PDMS could be estimated as the temperature change of perovskite layer. Low specific heat capacity, decreased film thickness, and limited heat loss at the interface is beneficial for the temperature rise and thus the final acoustic pressure. We experimentally measured the specific heat capacity for several typical materials utilized in optoacoustic transducers. As shown in Fig. 2b, the specific heat capacity is ~308 J kg^−1^ K^−1^ for perovskite ranging from 280 to 400 K, and this is only half or 1/5 of the carbon-based materials (candle soot particles: ~750 J kg^−1^ K^−1^; double-walled CNTs: ~1600 J kg^−1^ K^−1^).

In terms of the heat loss, we take the view that the thermal diffusion within the absorbers plays a major role in transferring heat to the far point from PDMS, and the ideal absorber should have low thermal diffusion coefficient. The thermal diffusion coefficient was measured by the laser thermal conductivity meter. Figure 2c and Supplementary Fig. 4 show that MAPbI_3_ has a low thermal diffusion coefficient of 0.145 mm^2^ s^−1^, much smaller than other carbon materials (carbon black: 0.314 mm^2^ s^−1^; carbon nanofibers: 0.342 mm^2^ s^−1^; double-walled CNTs: 1.165 mm^2^ s^−1^; multi-walled CNTs: 0.678 mm^2^ s^−1^).

According to above measurement results, we could roughly derive the temperature rise (Δ*T*) at the interface between MAPbI_3_ and PDMS as 292 K given the laser pulse of 3 mJ, while the detailed calculation process is shown in Supplementary Note 3. In contrast, the temperature rise (Δ*T*) is only 38 K for candle soot particles. The ratio of Δ*T* for candle soot particles and MAPbI_3_ (38/292 = 0.13) is very close to the ratio of the measured optoacoustic conversion efficiency (0.15), where the conversion efficiency of MAPbI_3_ is 2.97 × 10^−2^ and that of candle soot particles is 0.441 × 10^−2^ (ref. ^24^). Such consistency verifies the correctness of our analysis that the low specific heat capacity, small thermal diffusion coefficient and high absorption coefficient of MAPbI_3_ are the key for obtaining high peak-to-peak acoustic pressure and optoacoustic conversion efficiency.

To further understand the origin of the low thermal diffusion coefficient of MAPbI_3_, we conducted density functional theory (DFT) calculations. Principally, three distinct relaxation stages exist during heat transport as shown in Fig. 2d: (1) carrier-phonon scattering (Fröhlich interaction, process ①); (2) optical phonon decay to acoustic phonons (process ②); and (3) acoustic phonon propagation to the far-field region in the materials (thermal conduction, process ③). The acoustic phonons dominate the heat transport by lattice vibrations. The calculated phonon dispersion relations and full phonon density of states (DOS) of MAPbI_3_ are shown in Fig. 2e, f, respectively. The acoustic bands are labeled with red colors. The negative frequency is attributed to the metastable lattice of MAPbI_3_, where the distortion of the crystal lattice could contribute to the permanent atomic displacement. Evidently, no complete phonon band gap is observed, and there is overlap between the optical and acoustic phonons. In the partial phonon DOS (see Fig. 2f), the low-frequency modes (acoustic modes) mainly come from PbI_3_^−^, which is related to the motion of the whole Pb–I lattice. On the other hand, the high frequency modes (>3 THz) consist entirely of the contributions from CH_3_NH_3_^+^ cations. Considering the non-propagating vibrations of organic cations, these modes have a relatively flat band dispersion (see Fig. 2e). Furthermore, there is a significant coupling between PbI_3_^−^ and CH_3_NH_3_^+^ components in the low-frequency range ~0–3 THz and the phonons are called “hybrid phonons”. The organic–inorganic hybrid compositions of MAPbI_3_ facilitates the up-conversion of acoustic phonons to optical phonons, and thus hot phonon bottleneck effect. The overlap between optical and acoustic modes has also been elaborated in previous studies^12,31,32^. Claudio Quarti and coworkers have reported the low-frequency resonant Raman spectrum of MAPbI_3_. The bands at 62 and 94 cm^−1^ are assigned, respectively, to the bending and to the stretching of Pb–I bonds, and the bands at 119 and 154 cm^−1^ are assigned to the librations of the MA^+^ (ref. ^31^). The even lower bands (<50 cm^−1^) is ascribed to the “hybrid phonons”. Gavin Conibeer and coworkers further studied the phonon spectrum of hybrid perovskite and inorganic perovskites. They also found that the series of low-energy optical modes (30–100 cm^−1^, equal to 3.72–12.4 meV) are ascribed to the coupling of the libration and torsion of MA^+^ and the lead iodide lattices, while these low-energy modes is the above-mentioned “hybrid phonons”. In contrast, they found in inorganic perovskite (CsPbIBr_2_), there is reduced phononic density of states, and the carrier relaxation rate is also faster than organic–inorganic hybrid perovskite^12^. Thereby, we believe the origin of the overlap of optical and acoustic phonons is unique property for organic–inorganic hybrid perovskites, and the co-vibrations between the organic and inorganic sub-lattices contribute to the overlap optical phonons. As a result, the probability of acoustic phonons scattering to optical states and recycle the vibrational energy is increased. The efficient acoustic phonon up-conversion can recycle the thermal energy, reheat the carriers and lead to the rather low thermal diffusion coefficient (see Fig. 2d).

We further studied the photostriction, lattice expansion and polarization effect of perovskite under photoexcitation, to exclude the influence of other photoexcitation effect on the observed optoacoustic phenomenon. The linear expansion (*dL/L*) caused by photostriction of MAPbI_3_ is ~4 × 10^−5^ (ref. ^33^), which is three orders smaller than the thermal expansion of PDMS (*dL/L* = 3.2 × 10^−4^ K^−1^ × 292 K= 9.3 × 10^−2^)^34^. The linear thermal expansion coefficient of MAPbI_3_ (3.9 × 10^−4^ K^−1^) is comparable to PDMS^35^, but considering the thickness difference of MAPbI_3_ (~300 nm) and PDMS (20 μm), the thermal expansion of MAPbI_3_ itself could be neglected. Moreover, previous studies demonstrate that the polaron under photoexcitation and photon bottleneck effect could both slow down the hot-carrier cooling process. But the polaron effect is only dominant under low excitation density (*n* ≤ 10^18^ cm^−3^) for MAPbI_3_; in contrast, under high excitation density (*n* ≥ 10^18^ cm^−3^), the phonon bottleneck effect plays the major role^36^. We could calculate the excitation density according to the following equation: $n=\frac{{E}_{{\mathrm{laser}}}h{c}_{0}}{\lambda {Ad}}$, where *E*_laser_ is the energy of the laser pulse, *h* is the Plank constant, *c*_0_ is the velocity of light, *d* is the thickness of MAPbI_3_ thin film (323 nm), *A* (0.0625 cm^2^) is the area of the laser spot, and *λ* (532 nm) is the center wavelength of the laser. Then we can derive the excitation density of MAPbI_3_ thin film from the equation and the excitation density is ~1.3 × 10^21^ cm^−3^ with 1 mJ laser pulse energy, which will lead to the phonon bottleneck effect rather than polaron effect.

### Performance optimization of the optoacoustic transducer

**Fig. 3:**
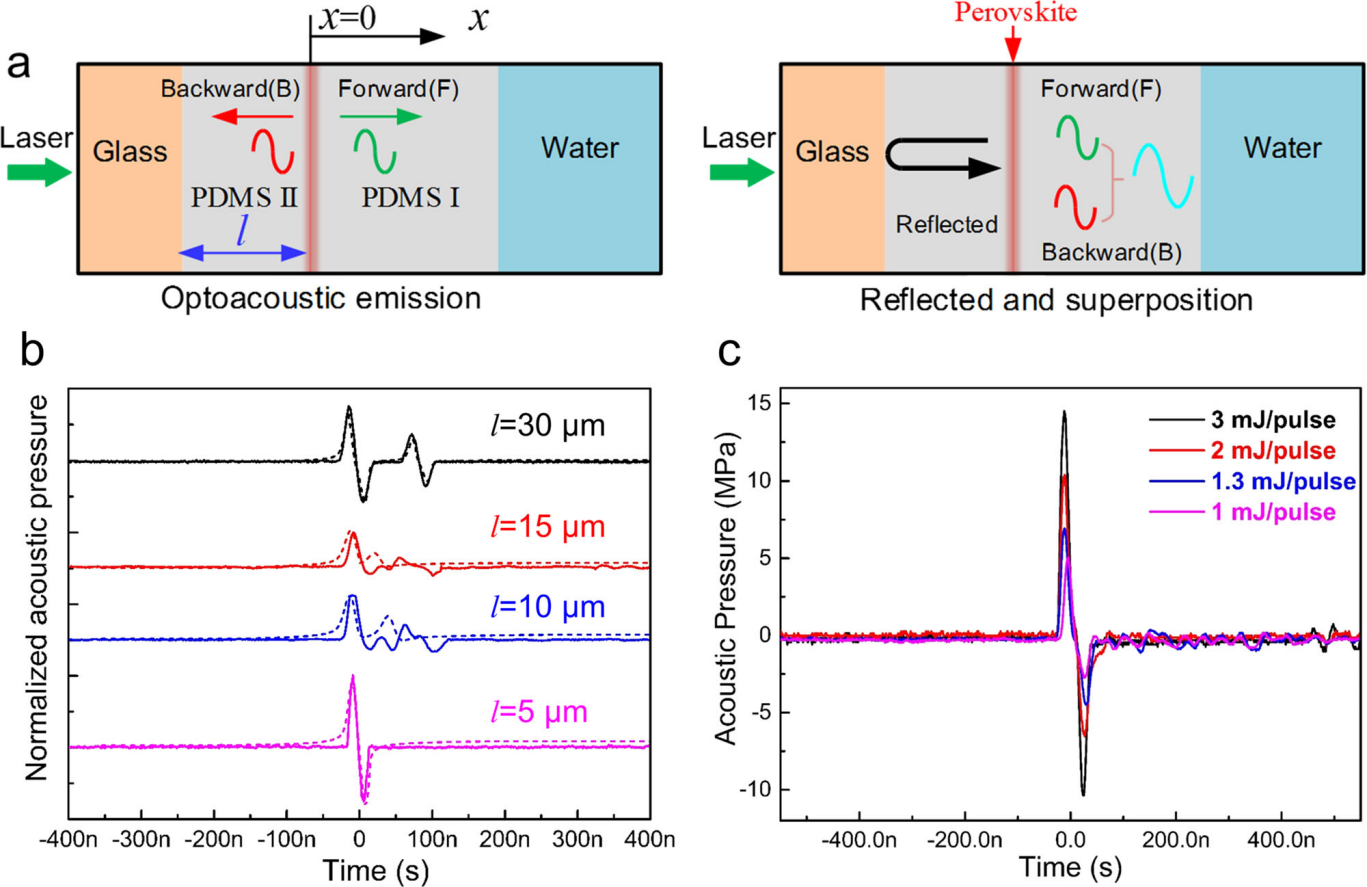
The influence of the thickness of PDMS layer on the wave propagation. **a** Schematic illustration of optoacoustic signal propagation. **b** Theoretical (dotted line) and experimental (solid line) results of acoustic pressure as the change of PDMS II layer thickness. The black, red, blue, and pink curves represent the acoustic pressure results for PDMS thickness of *l* = 30, 15, 10, and 5 μm, respectively. **c** The amplitude of optoacoustic pressure at different light intensities. The black, red, blue, and pink curves represent the acoustic pressure under the light intensity of 3, 2, 1.3, and 1 mJ pulse^−1^, respectively.

We optimize the optoacoustic transducers before assembling the device onto the fibers. As shown in Fig. 3a, optoacoustic signal is generated at the interface of PDMS and MAPbI_3_. PDMS I layer (thickness: *h*) protects MAPbI_3_ from degradation by water, and PDMS II layer can effectively prevent heat loss through the glass substrate and also contribute to the superposition of the ultrasound waves. The laser-induced ultrasound can simultaneously propagate forward and backward, as marked by F wave and B wave, respectively. The ratio of reflected acoustic pressure to incident acoustic pressure at the interface between PDMS and glass is *r*_*p*_, which can be expressed by the following equation^37^.

2 $${r}_{p}=\frac{{p}_{B}{\prime} }{{p}_{B}}=\frac{{R}_{2}-{R}_{1}}{{R}_{2}+{R}_{1}}=\frac{1-{R}_{1}/{R}_{2}}{1+{R}_{1}/{R}_{2}}$$

where *R*_1_ is the acoustic impedance of PDMS (1.5 × 10^5^ kg m^−2^ s^−1^), *R*_2_ is the acoustic impedance of glass (1.23 × 10^7^ kg m^−2^ s^−1^), the incident and reflected acoustic pressure are represented by *P*_B_ and *P*_B′_′, respectively. It can be seen that the magnitude of acoustic pressure reflected at the interface depends on the acoustic impedance of the medium. For the hard glass substrates, *R*_2_ ≫ *R*_1_, and *r*_*p*_ ≈ 1. Therefore, the B wave would be totally reflected at the glass interface, and the polarity of reflected sound wave would not change (see Supplementary Note 4)^37–40^.

In our design, the thickness of PDMS II is at the order of micrometers, and the velocity of the ultrasound propagation in PDMS is 1076 m s^−1^, so the delayed time between B wave and F wave is estimated as ~ns. The recorded ultrasound signal is believed to be the superposition of two acoustic waves, as shown in Fig. 3a. Clearly, the PDMS II layer is crucial, because its thickness determines the delayed distance of B wave and F wave^28,38^, and influence the superposition effect of two waves. Since the acoustic impedance of PDMS is similar to that of water, the reflection of sound wave at the interface between PDMS and water is negligible. Also, the thickness of perovskite is much smaller than acoustic wavelength (*λ* ≈ 36 μm) in PDMS, its influence on the ultrasound delay can be ignored. The analytical solution of the superposed acoustic pressure of F wave and B wave in water (*x* ≥ *h*) can be expressed by the following equation (see Supplementary Note 4)^38,41^.

3 $$P(x,t)=D({e}^{j\omega t-{kx}}+{e}^{j\omega (t+2l/{c}_{1})-{kx}})$$

where *D* is the constant related to the light absorption and thermal parameters of the material; *k* = *jω*/*c*, *c* is the sound speed in water, *c*_1_ is the sound speed in PDMS, *l* is the thickness of PDMS II layer, *ω* is the angular frequency, *t* is the time, and *x* is the coordinate. As shown in Fig. 3b and Supplementary Fig. 7, when the value of *l* changes, different sound waveform can be obtained theoretically and experimentally. It can be found that the waveforms and acoustic pressure amplitude of the measured results agree well with the simulated results. For optoacoustic transducer, when *l* = 5 μm, 2*l* < *λ*/2, 2*l*/*c*_1_ < *T/*2 (*T* = 1/*f*, *f*: ultrasound frequency), superposition effect of F wave and B wave is the strongest to form unipolar wave. However, when *l* = 10 μm, 15 μm, *λ*/2 < 2*l* < *λ*, *T*/2 < 2*l/c*_1_ < *T*, the superposition of F wave and B wave is weakened and no unipolar wave was formed; when *l* = 30 μm, *λ* < 2*l*, *T* < 2 *l/c*_1_, F wave and B wave are separated, so there is no superposition (see Supplementary Note 4). Simultaneously, the PDMS II layer can effectively reduce heat loss through the glass substrate, which is shown in Supplementary Fig. 9, thereby improving the acoustic pressure and optoacoustic conversion efficiency (*η*)^42^. When *l* = 0 μm, the peak-to-peak acoustic pressure is 14.14 MPa (laser energy: 3 mJ pulse^−1^, positive acoustic pressure: 9.07 MPa), *η* = 0.84 × 10^−2^. When *l* = 5 μm (negligible heat loss), the peak-to-peak acoustic pressure reaches 24.89 MPa (laser energy: 3 mJ pulse^−1^, positive acoustic pressure:14.52 MPa), *η* = 2.97 × 10^−2^ (see Fig. 3c).

### Application of the optical fiber-based optoacoustic transducer

Actually, miniature ultrasound device is of great importance for biomedical applications, especially for invasive ultrasound diagnosis and therapy^43^. In order to realize the miniaturization of the optoacoustic transducer structure, we coated MAPbI_3_ onto the fiber-optic end surfaces by dipping the fibers into the precursor solution and subsequent drying at 120 °C for 10 min. The size comparison between our fiber-optic optoacoustic transducers and commercial piezoelectric hydrophone (Precision Acoustic, UK) is shown in Supplementary Fig. 10. The smallest diameter of commercial needle hydrophones is 200 μm. Obviously, our fiber-optic optoacoustic transducers can be even smaller in size (125 μm), and has no difficult electrical connection and electromagnetic interference.

**Fig. 4:**
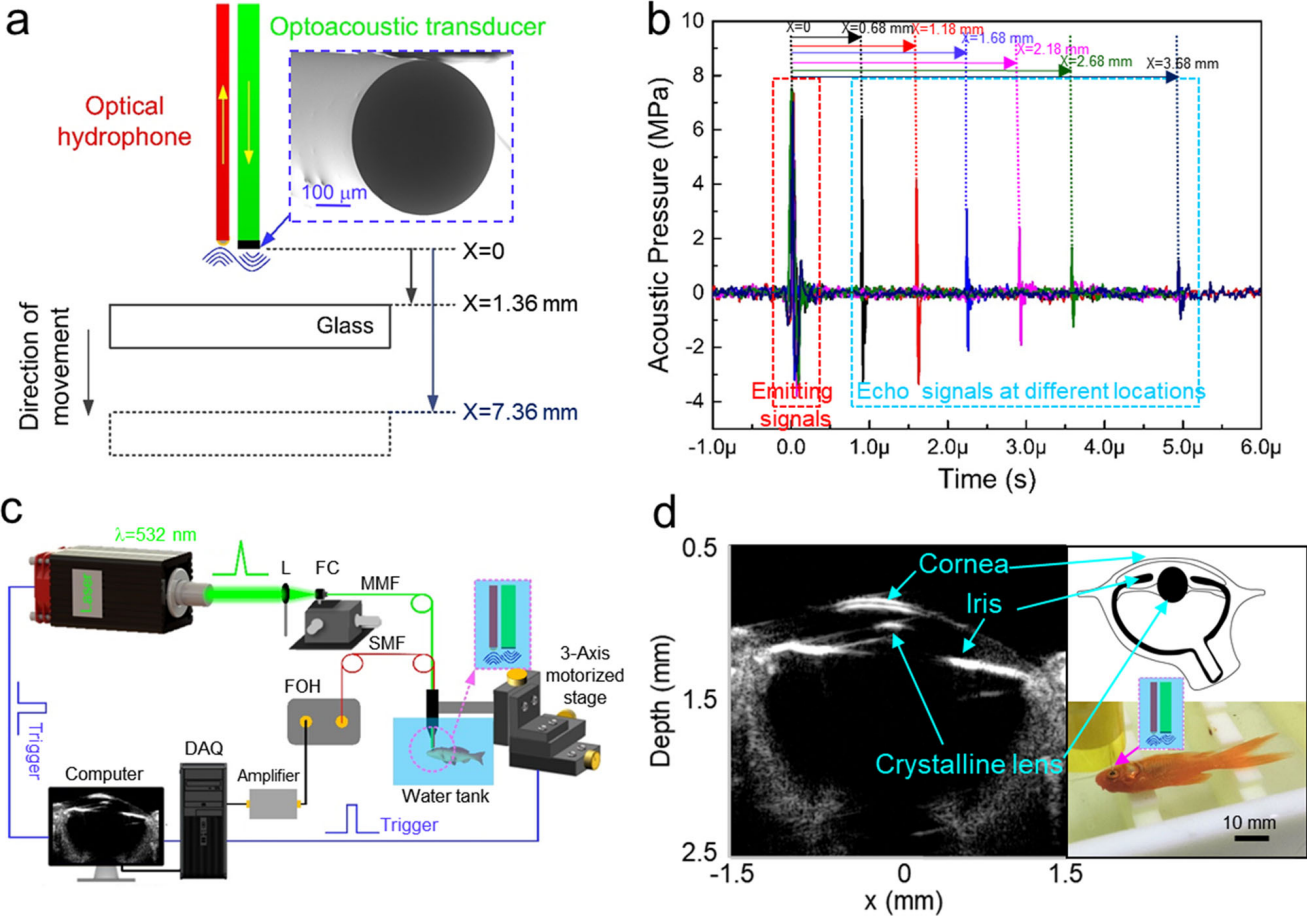
The optical fiber-based optoacoustic transducer. **a** Schematic diagram of echo signal detection. **b** Measured echo signals reflected by glass at different locations. The gray, red, blue, pink, green, and dark blue curves represent the echo signals reflected by the glass at *x* = 0.68, 1.18, 1.68, 2.18, 2.68, and 3.68 mm, respectively. **c** The all-optical ultrasound imaging system. L lens, FC fiber coupler, MMF multimode fiber, SMF single-mode fiber, FOH fiber-optic hydrophone, DAQ data acquisition card. **d** Laser-generated ultrasound for imaging of fisheye.

To avoid coffee ring effect at the fiber end, we added polyvinylpyrrolidone (PVP) into the precursor solution for decreasing the crystallization rate, while the optimized concentration is 15 wt% (see Supplementary Fig. 11). The smooth end surface of fiber-optic coated with PDMS/MAPbI_3_ and PDMS/MAPbI_3_/PDMS are shown in Supplementary Fig. 12a, b, respectively. The coated film has uniform thickness^44^. Fig. 4a presents the schematic diagram of all-optical ultrasound echo detection. Laser-generated ultrasound is emitted by the fabricated fiber-based optoacoustic transducer, and signal is received by commercial optical hydrophone (diameter: 125 μm). Both of them are parallelly integrated to a probe. In Fig. 4b, the first signal is the transmitting signal and the other signals are ultrasound echoes reflected by glass at different locations. Thereby, according to the arrival time of the signals, we could obtain the spatial information. Based on this principle, an ultrasound imaging system is set up, as shown in Fig. 4c. Controlled by a commercial LabVIEW program, a motor drives the probe to carry out the scanning procedure. FPGA is employed to generate corresponding trigger signals to realize timing synchronization and a data acquisition card at a sampling rate of 250 MS s^−1^ (12 bit; ATS9325, Alazar) is utilized for digitizing echo signals. The longitudinal resolution of ultrasound imaging is determined by the bandwidth of the optoacoustic transducer^45^. Here, we obtain the full-width at half-maximum (FWHM) of the enveloped line of the optoacoustic wave as the longitudinal resolution. As shown in Supplementary Fig. 13, the FWHM of the obtained transducer is ~36 μm. Figure 4d describes the ultrasound imaging of the fisheye with high resolution and good signal-to-noise ratio. Obviously, the structure of cornea, iris, and lens surface can be clearly resolved. Mostly importantly, the cornea’s thickness can be determined as 56 μm, which is consistent with the documented results^46^. This is mainly attributed to that the ultrasound produced by MAPbI_3_-based device has high operational frequency (~30 MHz) and its high conversion efficiency guarantees the strong ultrasound intensity.

## Discussion

In summary, we demonstrate the combination of lead halide perovskites and PDMS produces excellent optoacoustic transducers with broad bandwidths (40.8 MHz), large acoustic pressure (peak-to-peak: 24.89 MPa), and high conversion efficiency (2.97 × 10^−2^). Such good performance benefits from the large absorption coefficient, low specific heat capacity (~308 J kg^−1^ K^−1^), and low thermal diffusion coefficient of MAPbI_3_. We theoretically verify that the overlap of organic cations-related optical phonons and inorganic skeleton-related acoustic phonons is the origin of low thermal conductivity. Moreover, the convenient fabrication of perovskite films onto the small fibers enables the use of optoacoustic transducers as miniaturized ultrasound source, and achieves a high-resolution ultrasound imaging of fisheye. Our work shows an encouraging case of utilizing the unique thermal related properties of metal halide perovskites. This work also provides a paradigm for designing efficient optoacoustic transducers, and pushes this technology closer to the practical applications, including biomedical imaging, therapeutic ablation, and brain modulations.

## Methods

### Materials

Methylamine solution (Aladdin, ~30 wt% in hydroiodic acid, ≥47.0%). PDMS (SYLGARD-184) base and curing agent were purchased from Dow corning. Lead iodide (99.9%), diethyl ether (99%), DMSO (99%), and DMF (99%) were purchased from Sigma-Aldrich. The glass/ITO substrates (2.5 cm × 2.5 cm) were purchased from Kaivo Optoelectronic Technology Co., Ltd (Zhuhai, China) and cleaned sequentially by detergent, deionized water, acetone, isopropanol, and ethyl alcohol with the assistance of ultra-sonication for 15 min.

### Synthesis of CH_3_NH_3_I

Firstly, 50 mL methylamine solution and 50 mL ethanol were mixed together in the round bottom flask. Then, 60 mL hydroiodic acid (≥47.0% wt) together with 1 mL hypophosphorous acid were loaded in a separatory funnel, and added drop wise into the flask. The dropping process took ~20 min, and the mixture was stirred for 3 h for complete reaction. Subsequently, the flask was immersed in a water bath at 65 °C, and the solution was evaporated in a rotary evaporator. The crude product was washed with a large amount of diethyl ether, and was then dissolved in a beaker with 10 mL ethanol by heating to proper temperature. Next, the mixture was slowly cooled to 4 °C for recrystallization. Finally, the white crystalline product MAI was dried under vacuum at 60 °C for 24 h.

### Preparation of MAPbI_3_ solution

A total of 2.305 g PbI_2_ (5 mmol) and 0.79 g MAI (5 mmol) were dissolved in 3.5 mL DMF and 1.5 mL DMSO in a glass bottle.

### Preparation of PDMS precursor

A total of 5 g PDMS base and 0.5 g curing agent were mixed evenly in a small beaker, and then placed in a vacuum drying oven to degas for 30 min.

### Fabrication of the optoacoustic transducer

First, the PDMS precursor was spin-coated onto the glass substrates or the concave glass substrate, and then placed on the hot plate for ~20 min at 120 °C. Then the PDMS substrate was treated by 5 min ozone plasma to be hydrophilic. Next, the as-prepared MAPbI_3_ perovskite solutions were coated onto the PDMS substrate by a spin-coating process at 300 r.p.m. for 3 s and 2500 r.p.m. for 60 s in a glovebox. At the 52 s of the second-step spin-coating, the substrate was treated with 700 μL diethyl ether antisolvent drop-casting. The obtained samples were thermally annealed on a hot plate for 10 min. Finally, another PDMS layer was spin-coated onto the MAPbI_3_ layer.

### Fabrication of optical fiber-based optoacoustic transducer

A 15 wt% PVP was added into perovskite precursor solution. Firstly, the fiber end was immersed in the perovskite precursor solution, and held stationary for 15 s, and subsequently withdrawn at a rate of 6 cm min^−1^. The optical fiber was annealed in an oven at 120 °C for 10 min to obtain the perovskite film. Then the perovskite-coated fiber was again immersed into PDMS solution (the mass ratio of PDMS to toluene is 1:1), held stationary (10 s), and subsequently withdrawn at a rate of 6 cm min^−1^. The fiber end was kept upward, held at room temperature for 30 min, and dried at 100 °C for 20 min.

### Material characterizations

The phase purity of MAPbI_3_ thin film was identified by X-ray diffraction equipment (Philips, X pert pro MRD, Cu Kα radiation, *λ* = 1.54178 Å). The morphology was studied by SEM (FEI Nova NanoSEM450, without Pt coating). The absorption spectra of devices were measured by UV–vis spectrophotometer (PerkinElmer Instruments, Lambda 950) with an integrating sphere. The thermal diffusion coefficient of the light absorbing material was evaluated by using a laser thermal conductivity meter (LFA467, Netzsch, Germany). The specific heat capacity of light absorbing material was characterized by employing a physical property measurement system (PPMS-9T, Quantum Design, USA).

### Device characterization

A pulsed laser (*λ* = 532 nm, repetition rate: 20 Hz, pulse width: 6 ns, Lapa-80, Bejing LeiBao Optoelectronics Technology Co., Ltd) was used as the light source. The laser beam (spot size: 5 mm) penetrated through a transparent wall of the water tank, illuminating the photoacoustic transducer. The acoustic wave was detected by operating a fiber-optic hydrophone (diameter: 125 μm, FOH, Precision Acoustic, UK) with the frequency range of 250 kHz–50 MHz and pressure ranges of 10 kPa–15 MPa; the resulting signal was recorded by an oscilloscope (TDS-2024B, Tektronix, USA). The distance between the hydrophone end and the photoacoustic transducer is 2 mm.

### Computational methods

DFT calculations were carried out using the Vienna Ab initio Simulation Package (VASP 5.4.4)^47,48^. A plane-wave basis set with 500 eV cutoff energy was selected to expand the wave functions. For the exchange-correlation functional, we adopted the generalized gradient approximation, under the Perdew–Burke–Ernzerhof for solids functional form (PBEsol)^49^. The electronic configurations are 1s for H, 2s and 2p for C, 2s and 2p for N, 6s and 6p for Pb, and 5s and 5p for I. Projector augmented-wave pseudopotentials^50,51^ were used to replace the core electrons. Sufficiently dense equal-spacing *k*-point meshes^52^ were utilized to sample the Brillouin zones, i.e., 5 × 4 × 5 for structural relaxation. During structural optimization, the total energy convergence criterion was set to <10^−7^ eV, while the force criterion was set to 0.001 eV Å^−1^. The phonon dispersion relations were calculated with the assistance of the PHONOPY code^53^.

The fish used in our experiment is a goldfish which belongs to *Carassius auratus*. The size of the fish is ~50 mm × 25 mm × 10 mm. We have complied with all relevant ethical regulations for animal testing and research and the ethical approval report is provided as a Supplementary file.

### Reporting summary

Further information on research design is available in the Nature Research Reporting Summary linked to this article.

## Supplementary information

Supplementary Information

Reporting Summary

## Data Availability

All data needed to evaluate the conclusions in the paper are present in the paper and in the Supplementary Information.
