# Supplementary material for: Lead halide perovskite for efficient optoacoustic conversion and application toward high-resolution ultrasound imaging
Source: Nat Commun. 2021 Jun 7;12:3348. doi: 10.1038/s41467-021-23788-4 (PMC8184828; doi:10.1038/s41467-021-23788-4)
Supplement: Supplementary file 1 — Supplementary Information [file 41467_2021_23788_MOESM1_ESM.pdf]

Supplementary Information for

Lead halide perovskite for efficient optoacoustic conversion and application toward high-resolution ultrasound imaging

Xinyuan Du[1,#], Jiapu Li[1,2,#,] Guangda Niu[1,*], Jun-Hui Yuan[1], Kan-Hao Xue[1], Mengling Xia[1], Weicheng Pan[1], Xiaofei Yang[1], Benpeng Zhu[1,2,*], Jiang Tang[1]

[1]Wuhan National Laboratory for Optoelectronics, School of Optical and electronic information, Huazhong University of Science and Technology, Wuhan, China, 430074

[2]State Key Laboratory of Transducer Technology, Chinese Academy of Sciences, Shanghai, 200050, China

[#]Xinyuan Du and Jiapu Li contribute equally to this paper

*corresponding author: Prof. Guangda Niu: guangda_niu@hust.edu.cn; Prof. Benpeng Zhu: benpengzhu@hust.edu.cn.

Supplementary Note 1. Multiphysics simulation.

The detailed conditions are as follows:

Frequency domain: f_0=30 MHz;

Unit size 1500/f_0/5 mm;

T_0=293.7 K;

The sound speed in the water:1500 m/s;

Boundary conditions: the glass substrate is an acoustic hard boundary, and water is a perfectly matched layer.

Supplementary Table 1 COMSOL simulation parameters

Geometric dimensioning		Glass	PDMS II	$MAPbI_3$	PDMS I
	Radius	2.5 mm	2.5 mm	2.5 mm	2.5 mm
	Thickness	1 mm	5 μm	0.5 μm	1 mm
Physical parameter		water	$MAPbI_3$	glass	PDMS
	β (K^{-1})	2.1×10^{-4}	1.17×10^{-4}	0.08×10^{-4}	9.6×10^{-4}
	κ (W m^{-1} K^{-1})	0.6	0.5	0.8	0.15
	C (J kg^{-1} $^{o}C^{-1}$)	4.2×10^{3}	308	840	1.38×10^{3}

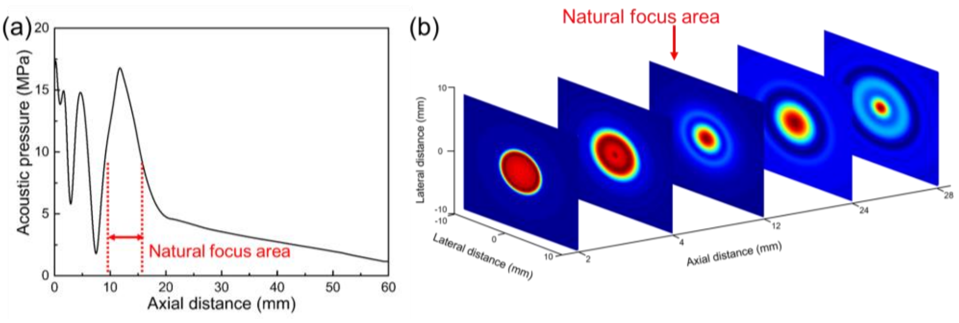

Supplementary Figure 1. (**a**) Acoustic pressure of the optoacoustic transducer calculated by COMSOL simulation; (**b**) the lateral sound field distribution of the optoacoustic transducer along the axial direction.

Supplementary Figure 1 (a) is the simulation result of the acoustic pressure of the optoacoustic transducer along axial direction. At 2 mm, the maximum acoustic pressure of COMSOL simulation is about 17 MPa, and the experimental result is around 15 MPa. The difference may be due to the neglect of the sound attenuation in the water. Supplementary Figure 1 (b) shows the lateral sound field distribution of the optoacoustic transducer along the axial direction. Its natural focal point is approximately $d^2/4\lambda$=12.5 mm (transducer diameter: d=5 mm, sound wave length: λ=50 μm). Within the focal distance, the ultrasound beam width nearly keeps the same, which is close equal to the size of the transducer (5 mm). Outside the focal distance, the ultrasound beam will become broader and broader.

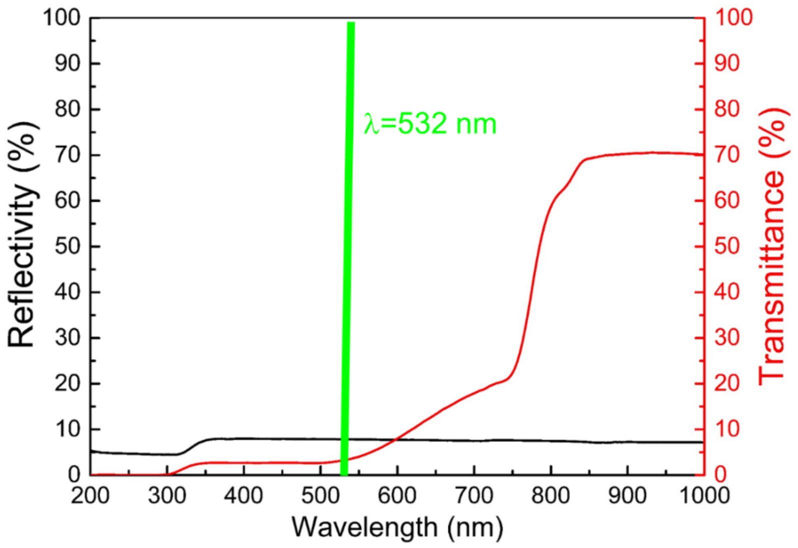

Supplementary Figure 2. Transmission and reflection spectra of $MAPbI_3$ film. When the laser wavelength is 532 nm, the light reflectance (R) and transmittance (T) are 7.85% and 3.31%, respectively.

Considering the reflection of the glass substrate and the transmission of the perovskite optoacoustic transducer (Supplementary Figure 2), the optoacoustic conversion efficiency can be expressed by the following equation.[1]

$$\eta = \frac{E_a}{(1-2R-T)E_{\mathrm{optical}}} \tag{S1}$$

$$E_a = \frac{1}{\rho c} A \int P^2(t)\mathrm{dt} \tag{S2}$$

where $P(t)$ is the acoustic pressure, A (1.96×10^{-5} m^2) is the area of laser facula, ρ (1×10^3 kg m^{-3}) is the density of water, c (1500 m s^{-1}) is acoustic speed in water, $E_{optical}$ (3 mJ) is the energy of pulse laser and E_a is the energy of acoustic , R (7.85%) is the reflectivity of the glass, T (3.31%) is the transmittance of perovskite photoacoustic transducer.

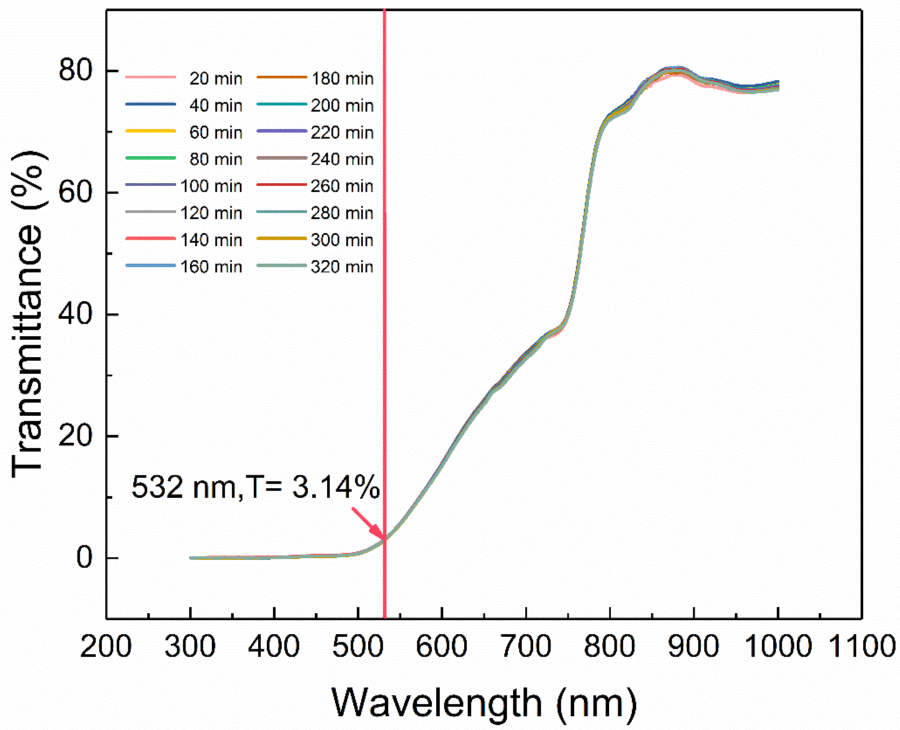

Supplementary Figure 3. The water stability of the optoacoustic device.

Supplementary Note 2. Theoretical calculation of the acoustic pressure

When the laser energy is 1 mJ, 1.3 mJ, 2 mJ and 3 mJ, the theoretical acoustic pressure values estimated (see below) are 2.98 MPa, 3.87 MPa, 5.96 MPa and 8.94 MPa, respectively. Taking into account the sound wave superposition enhancement caused by the PDMS II layer thickness of 5 μm, the superimposed acoustic pressures (Eq. (S3)) are 5.39 MPa, 6.99 MPa, 11.77 MPa and 16.16 MPa, respectively. The theoretical calculated value of acoustic pressure is more consistent with the experimental value (Supplementary Table 2), and the error may be caused by ignoring the sound wave attenuation.[2]

$$P=\sqrt{P_F^2+P_B^2+2P_FP_B\cos\theta} \tag{S3}$$

Where, P_B is the backward sound wave, P_F is the forward sound wave, neglecting the sound wave attenuation: $P_B = P_F$, θ is the phase difference between P_B and P_F.

Supplementary Table 2 Comparison of acoustic pressure theoretical calculation value and experimental test value.

Laser energy (mJ/pulse)	1	1.3	2	3
Experimental (MPa)	5.03	6.93	11.56	14.52
Theoretical (MPa)	5.39	6.99	11.77	16.16

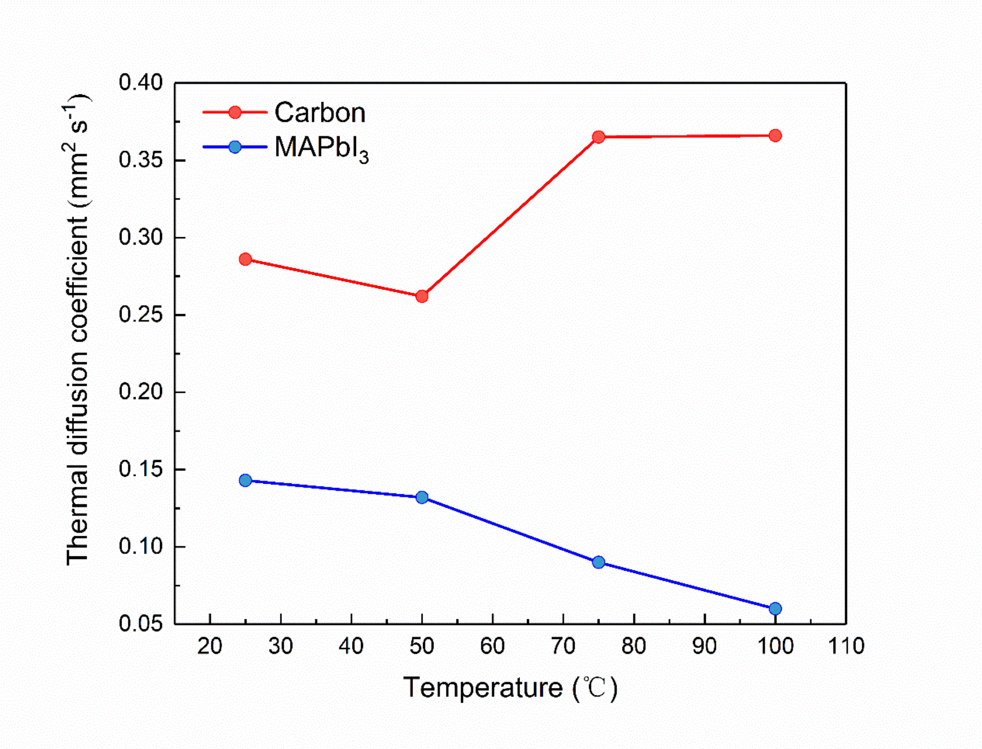

Supplementary Figure 4. Thermal diffusion coefficient of perovskite and traditional optoacoustic materials at different temperatures: The $MAPbI_3$ and carbon powder was pressed into wafer-like samples with a diameter of 12.7 mm and then we measured the thermal diffusion coefficient of the $MAPbI_3$ and carbon samples by a laser heat conductor. The thermal diffusion coefficient of the $MAPbI_3$ is 0.143 $mm^2\ s^{-1}$ at 25 ^oC, 0.132 $mm^2\ s^{-1}$ at 50 ^oC, 0.09 $mm^2\ s^{-1}$ at 75 ^oC and 0.06 $mm^2\ s^{-1}$ at 100 ^oC. The thermal diffusion coefficient of the carbon is 0.286 $mm^2\ s^{-1}$ at 25 ^oC, 0.262 $mm^2\ s^{-1}$ at 50 ^oC, 0.365 $mm^2\ s^{-1}$ at 75 ^oC and 0.366 $mm^2\ s^{-1}$ at 100 ^oC.

Supplementary Note 3. The calculation of temperature change(*ΔT*) of the interface between perovskite and PDMS.

Based on the assumption of local thermal equilibrium, the temperature change (*ΔT*) at the interface between perovskite and PDMS could be estimated as the temperature change of perovskite layer. Thereby ΔT can be described as: [3]

$$\Delta T = \frac{E_{optical}(1-e^{-\alpha d}) - H\rho Ad - Q_0}{C\rho Ad} \tag{S4}$$

where $E_{optical}$ is the energy of incident laser, α is the absorption coefficient, d is the film thickness ($MAPbI_3$: 323 nm), H is the latent heat coefficient of phase change ($MAPbI_3$: 4.16 J g^{-1} for the transition from tetragonal phase to cubic phase at 330.4 K), A is the area of laser facula (~0.196 cm^2), ρ is the absorber density ($MAPbI_3$: 4.16 g cm^{-3}), C is the specific heat capacity of the absorber layer, and Q_0 is the heat loss at the interface.

Supplementary Note 4. Analytical solution of acoustic pressure at different medium of optoacoustic transducer

In linear, non-viscous medium, the diffusion equation of heat wave can be expressed as: [1,4]

$$\kappa_i \nabla^2 T_i - \frac{\kappa_i}{\alpha_i}\frac{\partial T_i}{\partial t} = -S_i \tag{S5}$$

where κ, α, t, T, and S are the thermal conductivity, the thermal diffusivity, the time, the temperature, and an arbitrary thermal source, respectively. The PDMS I (thickness: h), perovskite (thickness: d) and PDMS II (thickness: l) are labeled as 1, 2 and 3, consecutively (Supplementary Figure 5).

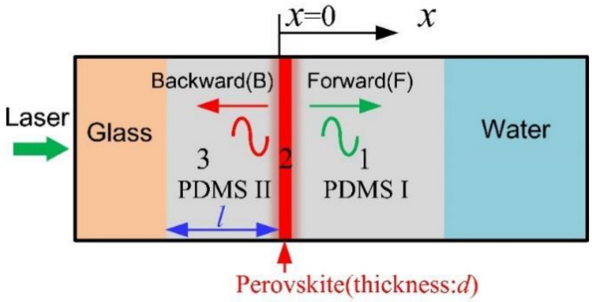

Supplementary Figure 5. Structure diagram of optoacoustic transducer.

Because the pulse width of the laser is ns level, the thermal diffusion during the acoustic generation can be neglected, yielding the equation,

$$\frac{\partial^2 T_i}{\partial t^2} = \frac{1}{\rho_i C_{P_i}}\frac{\partial S_i}{\partial t} \tag{S6}$$

where ρ is the density and C_P is the thermal capacity which equals $C_{P_i} = k_i/\rho_i \alpha_i$.

Therefore, the thermomechanical coupling equation in the medium can be expressed by the following equation.

$$\nabla^2 P_i - \frac{1}{c_i^2}\frac{\partial^2 P_i}{\partial t^2} = -\rho_i \beta_{T_i}\frac{\partial^2 T_i}{\partial t^2} \tag{S7}$$

where P is the pressure, β_T is the thermal volume expansion constant, and c is the acoustic speed in the medium.

Substituting the Eq. (S6) into Eq. (S7), We can obtain the equation of thermally induced mechanical vibration (pressure).

$$\nabla^2 P_i - \frac{1}{c_i^2}\frac{\partial^2 P_i}{\partial t^2} = -\frac{\beta_{T_i}}{C_{P_i}}\frac{\partial S_i}{\partial t} \tag{S8}$$

The spatial and temporal distribution of heat generated in the form of radiation-free transition after the heat source (2nd medium) absorbs light can be expressed as the following equation.

$$S_2 = \gamma I_0 e^{-\gamma x} e^{j\omega t} \tag{S9}$$

where γ is the light absorption coefficient, I_0 is the intensity of the incident light, ω is the angular frequency, x is the time, and x is the coordinate. The time-dependent harmonic pressure response can be expressed as $P_i = p_i e^{j\omega t}$, and the acoustic wave equation becomes

$$\frac{d^2 P_i}{dx^2} - k_i^2 P_i = -\frac{j\omega\beta_{T_i}}{C_{P_i}} S_i \tag{S10}$$

where $k_i = j\omega / c_i$, $S_2 = \gamma I_0 e^{-\gamma x}$ and $S_1 = S_3$.

The acoustic pressure amplitude in each medium can thus be solved as

$$\left.\begin{aligned} P_1 &= D_1 e^{-k_1(x-d)} \\ P_2 &= D_{21} e^{k_2 x} + D_{22} e^{-k_2 x} - \frac{j\omega\beta_{T_2}}{C_{P_i}}\left(\frac{\gamma I_0}{\gamma^2 - k_2^2} e^{-\gamma x}\right) \\ P_3 &= D_3 e^{k_3 x} \end{aligned}\right\} \tag{S11}$$

where D_1, D_{21}, D_{22} and D_3 are constants related to the optical and thermal properties of materials and can be determined by the boundary conditions. Due to the continuity of the acoustic medium at the interfaces 1 and 2, and 2 and 3, the acoustic pressure and sound velocity (amplitude) of the boundary medium can be expressed by the following equation.

$$\left.\begin{aligned} P_1\big|_{x=d} &= P_2\big|_{x=d} \\ P_2\big|_{x=0} &= P_3\big|_{x=0} \end{aligned}\right\} \tag{S12}$$

$$\left.\begin{aligned} v_1\big|_{x=d} &= v_2\big|_{x=d} \\ v_2\big|_{x=0} &= v_3\big|_{x=0} \end{aligned}\right\} \tag{S13}$$

with the pressure and the velocity related by $v_i = -(1/ j\omega\rho_i) dP_i / dx$. A set of equations for the undetermined constants is attained as

$$\begin{pmatrix} -1 & e^{k_2 d} & e^{-k_2 d} & 0 \\ 0 & 1 & 1 & -1 \\ \frac{k_1}{\rho_1} & \frac{k_2}{\rho_2}e^{k_2 d} & -\frac{k_2}{\rho_2}e^{-k_2 d} & 0 \\ 0 & \frac{k_2}{\rho_2} & -\frac{k_2}{\rho_2} & -\frac{k_3}{\rho_3} \end{pmatrix} \begin{pmatrix} D_1 \\ D_{21} \\ D_{22} \\ D_3 \end{pmatrix} = \begin{pmatrix} R_1 \\ R_2 \\ R_3 \\ R_4 \end{pmatrix} \tag{S14}$$

where

$$\left.\begin{aligned} R_1 &= \frac{j\omega\beta_{T_2}}{C_{P_2}}\left(\frac{\gamma I_0}{\gamma^2 - k_2^2}e^{-\gamma d}\right) \\ R_2 &= \frac{j\omega\beta_{T_2}}{C_{P_2}}\left(\frac{\gamma I_0}{\gamma^2 - k_2^2}\right) \\ R_3 &= -\frac{1}{\rho_2}\frac{j\omega\beta_{T_2}}{C_{P_2}}\left(\frac{\gamma^2 I_0}{\gamma^2 - k_2^2}e^{-\gamma d}\right) \\ R_4 &= -\frac{1}{\rho_2}\frac{j\omega\beta_{T_2}}{C_{P_2}}\kappa_2\left(\frac{\gamma^2 I_0}{\gamma^2 - k_2^2}\right) \end{aligned}\right\} \tag{S15}$$

It can be expressed by the following equation.

$$D_1 = \frac{Z_2\beta_{T_2}\gamma I_0}{\gamma^2 - k_2^2}\frac{\alpha_2}{\kappa_2}\frac{2(\gamma + k_2 R_{23}) - (\gamma + k_2)(1+R_{23})e^{(k_2-\gamma)d} - (\gamma - k_2)(1-R_{23})e^{-(k_2+\gamma)d}}{(1+R_{21})(1+R_{23})e^{k_2 d} - (1-R_{21})(1-R_{23})e^{-k_2 d}} \tag{S16}$$

Based on the equation above, the acoustic pressure at the PDMS I can be expressed as:

$$P_1(x,t) = D_1 e^{j\omega t - k_1(x-d)} \tag{S17}$$

Since the thickness of perovskite layer ($d \approx$ 300 nm) is much smaller than the wavelength of 30 MHz ultrasound ($\lambda \approx$ 36 μm) in PDMS, its influence on the ultrasonic delay can be ignored. Therefore, the forward-propagating (F wave) in PDMS I and back-propagating (B wave) in PDMS II can be expressed as:[5]

$$\left.\begin{aligned} P_F(x,t) &= D_1 e^{j\omega t - k_1 x} \\ P_B(x,t) &= D_1 e^{j\omega t + k_1 x} \end{aligned}\right\} \tag{S18}$$

The back-propagating (B wave) sound wave was totally reflected at the interface with the glass substrate after passing through the PDMS II layer. At this time, the sound pressure of the incident sound wave and the reflected sound wave have the same magnitude and the same polarity.

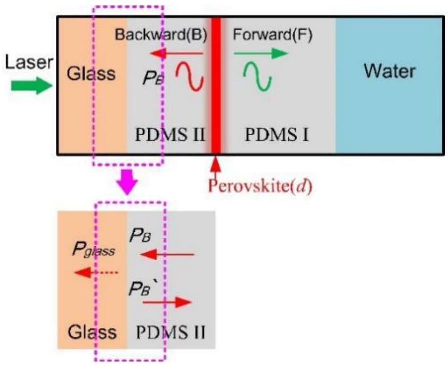

Supplementary Figure 6. Reflection and transmission of plane sound waves

Supplementary Figure 6 shows the reflection and transmission of sound waves at the acoustic boundary. The acoustic pressure in PDMS II is $P_B\,(x,\ t)$, and the acoustic pressure in glass is $P_{glass}\,(x,\ t)$. The magnitude of the reflected and transmitted acoustic pressure is now determined by the acoustic boundary conditions. The incident, reflected and transmitted acoustic pressure amplitude are represented by p_B, p_B` and p_{glass}, respectively. According to the acoustic boundary (x=0) conditions, the acoustic pressure should be continuous at the acoustic boundary.

$$P_{PDMS}(x,t)_{x=0} = P_{glass}(x,t)_{x=0} \tag{S19}$$

$$p_B = p_{glass} + p_B{}' \tag{S20}$$

The ratio of reflected acoustic pressure to incident acoustic pressure is r_p, which can be expressed by the following formula.

$$r_p = \frac{p_B{}'}{p_B} = \frac{R_2 - R_1}{R_2 + R_1} = \frac{1 - R_1 / R_2}{1 + R_1 / R_2} \tag{S21}$$

Where R_1 is the acoustic impedance of PDMS, R_2 is the acoustic impedance of glass. It can be seen that the magnitude of the acoustic pressure reflected at the interface depends on the acoustic impedance of the medium. For glass substrates (very hard), R2 >> R1, it can be obtained from Eq. (S21), $r_p \approx 1 > 0$. The results show that the $P_B\,(x,\ t)$ and the $P_B(x,t)'$ have the same amplitude and polarity.[2]

After the B acoustic wave is reflected by the glass, the acoustic pressure in PDMS II can be expressed as

$$P_B(x,t)' = P_B(-x, t + l / c_1) \tag{S22}$$

Therefore, F wave and B wave acoustic pressure in the PDMS I can be expressed as

$$\left.\begin{array}{l} P_F(x,t) = D_1 e^{j\omega t\text{-}k_1 x} \\ P_B(x,t)' = D_1 e^{j\omega(t+2l/c_1)-k_1 x} \end{array}\right\} \tag{S23}$$

At time t, the F wave and B wave at point x in PDMS I are overlay in time and space. According to the superposition principle, the synthesized acoustic pressure is expressed as:[6]

$$P_1(x,t)' = P_F(x,t) + P_B(x,t)' = D_1(e^{j\omega t-k_1 x} + e^{j\omega(t+2l/c_1)-k_1 x}) \tag{S24}$$

Ignore the attenuation of sound waves in PDMS and the difference with water, so acoustic pressure in water ($x \geqslant h$) can be expressed as:

$$P(x,t) = D(e^{j\omega t-kx} + e^{j\omega(t+2l/c_1)-kx}) \tag{S25}$$

Where $k=j\ \omega/\ c_{water}$, c_{water} (1500 m/s) is the speed of sound in the water, c_1 (1076 m/s) is the speed of sound in PDMS.

Where the function of PDMS II is to delay the B wave's, thereby controlling the superposition of the B wave and the F wave. The sound wavelength in PDMS can is about 36 μm ($f \approx 30$ MHz). When F wave and B wave delay distance $2l < \lambda/2 \approx 18$ μm, they can obviously be superposition enhancement (Supplementary Figure 7). When $l = 5$ μm, $2l < \lambda/2$, $2l/c_1 < T/2$ ($T=1/f$, f: ultrasound frequency), where F wave and B wave superposition effect is the strongest to form unipolar wave. However, when l=10 μm, 15 μm, $\lambda/2 < 2l < \lambda$, $T/2 < 2l/c_1 < T$, the superposition of F wave and B wave is weakened and no unipolar wave was formed; when l=30 μm, $\lambda < 2l$, $T < 2l/c_1$, F wave and B wave are separated, so there is no superposition.

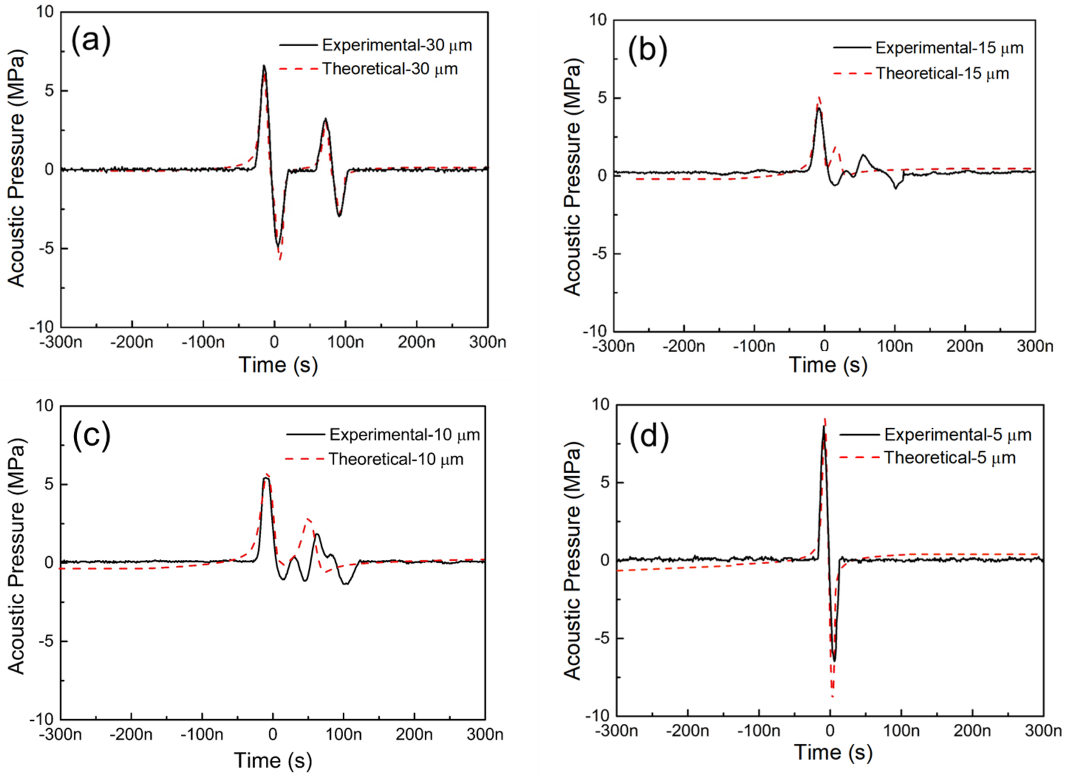

Supplementary Figure 7. Theoretical (red dotted line) and experimental (black solid line) results of acoustic pressure for PDMS II layer with different thickness, l=30 μm (**a**), l=15 μm (**b**), l=10 μm (**c**), and l=5 μm (**d**).

However, $2l$=0 is also superposition enhancement. But when l=0, the heat generated by the photothermal effect will be lost through the glass substrate, thereby reducing the optoacoustic conversion efficiency. There is a problem of thermal diffusion when heat is propagated in the medium, and the thermal diffusion length L can be expressed by the following equation:[7,8]

$$L = \sqrt{2\alpha\tau} \approx 40 \text{ nm} \tag{S26}$$

Where α (110 μm^2/s) is thermal diffusivity of PDMS, τ (6 ns) is the duration of pulse laser. It can be known that the $l >> L$, and the heat decays exponentially in the PDMS, almost no heat is lost from the glass substrate. When l=5 μm, the peak to peak acoustic pressure is 24.89 MPa (3 mJ/pulse), η=2.97×10^{-2}. When l=0, the peak to peak acoustic pressure is 14.14 MPa (3 mJ/pulse), η=0.84×10^{-2}.

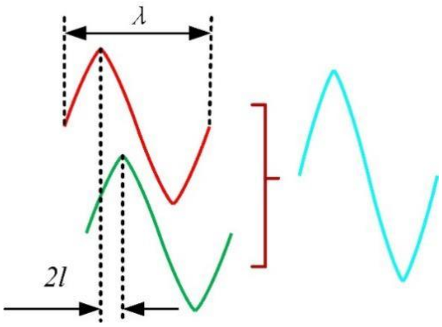

Supplementary Figure 8. Schematic diagram of sound wave superposition.

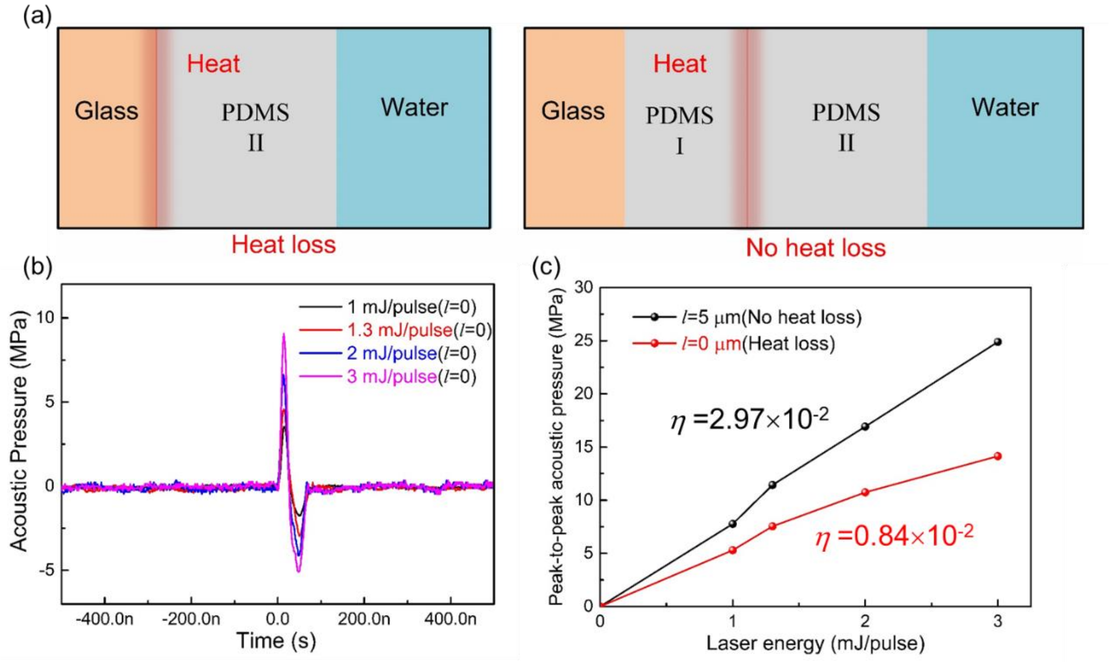

Supplementary Figure 9. Effect of PDMS I (*l*) layer on photo thermal loss. (**a**) Schematic diagram of the process of heat loss. (**b**) When $l = 0$, the acoustic pressure under different laser energy. (**c**) Peak-to-peak acoustic pressure and optoacoustic conversion efficiency under different laser energies (black line l=5 μm, red line l=0 μm).

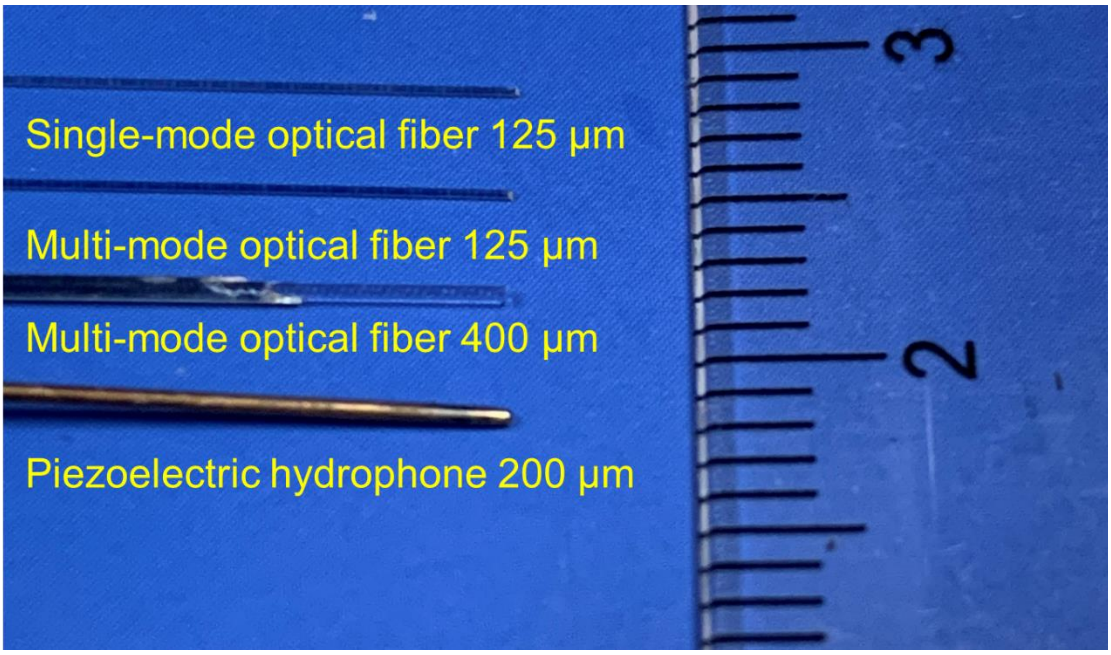

Supplementary Figure 10. Comparison of different ultrasonic transducer sizes.

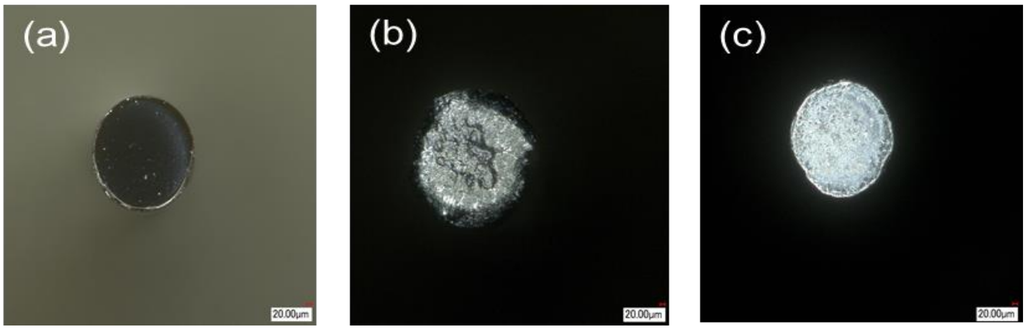

Supplementary Figure 11. The optical images of the end face of the optical fiber: Applying nothing (**a**), pure perovskite (**b**) and perovskite/15% PVP (**c**) to the fiber end face.

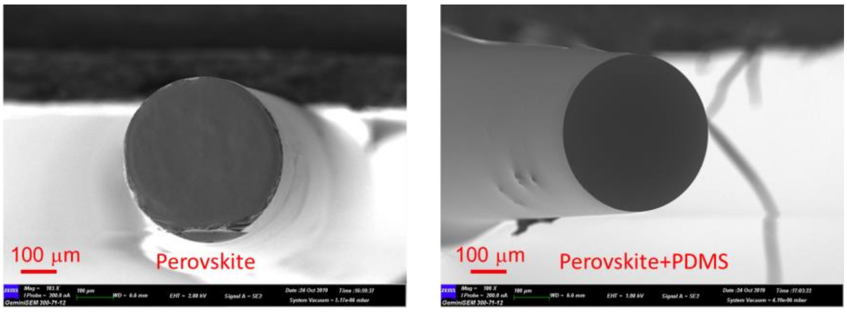

Supplementary Figure 12. SEM images of $MAPbI_3$ (**a**) and $MAPbI_3$/PDMS (**b**) at the fiber end face.

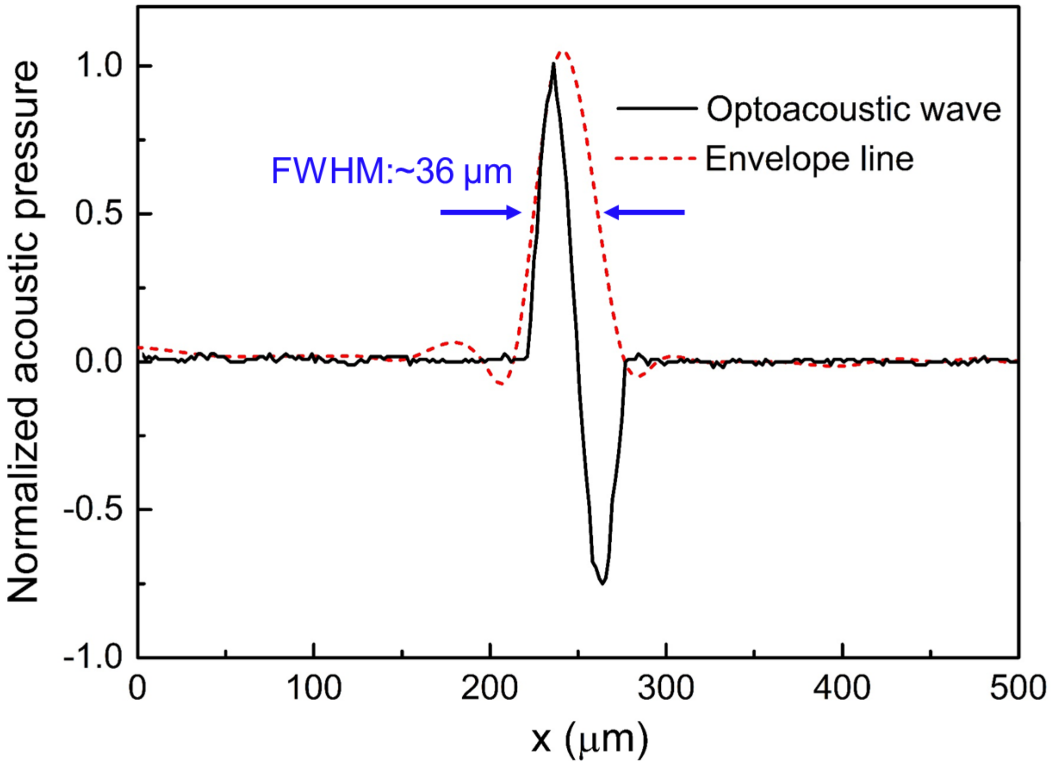

Supplementary Figure 13. The longitudinal resolution is given by enveloped axial line spread function (red dashed curve) of the optoacoustic wave (black solid curve).

The longitudinal resolution of ultrasound imaging is determined by the bandwidth of the optoacoustic transducer, as documented in previous studies.[9-11] As stated by Paul C. Beard and co-workers, the longitudinal resolution could be evaluated through the enveloped axial line spread function of the optoacoustic wave.[4] The enveloped longitudinal line full width at half-maximum (FWHM) is ~36 μm, as shown in Supplementary Figure 13.

References

[1] W. Huang, W. Chang, J. Kim, S. Li, S. Huang, X. Jiang. A novel laser ultrasound transducer using candle soot carbon nanoparticles. *IEEE T. Nanotechnol.* **15**, 395-401 (2016).

[2] J. Li, Y. Yang, Z. Chen, et al. Self-healing: A new skill unlocked for ultrasound transducer. *Nano Energy.* **68**, 104348 (2020).

[3] A. C. Tam. Applications of photoacoustic sensing techniques. *Rev. Mod. Phys.* **58**, 381 (1986).

[4] Z. Chen, Y. Wu, Y. Yang, et al. Multilayered carbon nanotube yarn based optoacoustic transducer with high energy conversion efficiency for ultrasound application. *Nano Energy.* **46**, 314-321 (2018).

[5] Du H, Zhu Z, Gong X. Acoustic basis. *Nanjing University Press*. *China* 201-205 (2001).

[6] T. Lee, Q. Li, L. J. Guo. Out-coupling of longitudinal photoacoustic pulses by mitigating the phase cancellation. *Sci. Rep.* **6**, 21511 (2016).

[7] S. M. Menke, R. J. Holmes. Exciton diffusion in organic photovoltaic cells. *Energy Environ. Sci.* **7**, 499-512 (2014).

[8] K. A. Brown, D. J. Eichelsdoerfer, W. Shim, et al. A cantilever-free approach to dot-matrix nanoprinting. *Proc. Natl. Acad. Sci.* **110**, 12921-12924 (2013).

[9] W. Xing, L. Wang, K. Maslov, L. V. Wang. Integrated optical- and acoustic-resolution photoacoustic microscopy based on an optical fiber bundle. *Opt. Lett.* **38**, 52-54 (2013).

[10] J. Shi, T. T. W. Wong, Y. He, et al. High-resolution, high-contrast mid-infrared imaging of fresh biological samples with ultraviolet-localized photoacoustic microscopy. *Nat.Photonics* **13**, 609–615 (2019).

[11] R. Ansari, E. Z. Zhan, A. E. Desjardins, P. C. Beard. All-optical forward-viewing photoacoustic probe for high-resolution 3D endoscopy. *Light Sci. Appl.* **7**, 75 (2018).
